# Increased Paternal Age at Conception Is Associated with Transcriptomic Changes Involved in Mitochondrial Function in Elderly Individuals

**DOI:** 10.1371/journal.pone.0167028

**Published:** 2016-11-23

**Authors:** Tapio Nevalainen, Laura Kananen, Saara Marttila, Juulia Jylhävä, Marja Jylhä, Antti Hervonen, Mikko Hurme

**Affiliations:** 1 Department of Microbiology and Immunology, School of Medicine, University of Tampere, Tampere, Finland; 2 Gerontology Research Center, Tampere, Finland; 3 School of Health Sciences, University of Tampere, Tampere, Finland; 4 Fimlab Laboratories, Tampere, Finland; Hokkaido Daigaku, JAPAN

## Abstract

The increased paternal age at conception (PAC) has been associated with autism spectrum disorder (ASD), schizophrenia and other neurodevelopmental disorders, thus raising questions that imply, potential health concerns in the offspring. As opposed to female oogonia, the male germ cells undergo hundreds of cell divisions during the fertile years. Thus, the advanced paternal age is associated with increase of point mutations in the male spermatogonia DNA, implying that this could be the major driving mechanism behind the paternal age effect observed in the offspring. In addition to replication errors, DNA replication fidelity and inefficient DNA repair machinery in the spermatogonia also contribute to the mutagenic load. Our study population consisted of 38 nonagenarians, participants in the Vitality 90+ Study, born in the year 1920 (women n = 25, men n = 13), for whom the parental birth dates were available. The gene expression profile of the study subjects was determined with HumanHT-12 v4 Expression BeadChip from peripheral blood mononuclear cells. We used Spearman's rank correlation to look for the associations of gene expression with paternal age at conception. Associated transcripts were further analyzed with GOrilla and IPA to determine enriched cellular processes and pathways. PAC was associated with the expression levels of 648 transcripts in nonagenarian subjects. These transcripts belonged to the process of mitochondrial translational termination and the canonical pathway of *Mitochondrial dysfunction*, more specifically of *Oxidative phosphorylation*. The observed systematic down-regulation of several mitochondrial respiratory chain components implies compromised function in oxidative phosphorylation and thus in the production of chemical energy.

## Introduction

The average age of childbearing in women and fathering in men is showing an increasing trend in developed countries due to recent changes in social and economic conditions in the past decades [1]. This delay in parenting is considered to be a potential public health concern because advancing parental ages at the time of conception are shown to be associated with adverse effects in the offspring.

In humans, the association of increased PAC with adverse effects was first reported in 1955 in the case of the genetic disorder achondroplasia [2]. More recently, advanced PAC has been linked to increased risk of miscarriage [3], pre-eclampsia [4], pre-term birth, low birth weight and fetal death [5]. Additionally, advanced PAC has been associated with a variety of psychiatric disorders in the offspring. These include schizophrenia [6], bipolar disorder [7], reduced neurocognitive abilities in childhood and infancy [8] and autism spectrum disorder (ASD) [9, 10]. Despite all the downsides, it has been speculated that effects associated with paternal age at conception are not necessarily restricted to adverse ones, because increased leukocyte telomere length in offspring has been reported in the offspring of older fathers [11]. Although the results concerning the effects of paternal age are ambiguous, the age of sperm donors is commonly advised not to exceed 40 years [12].

The common mechanism behind advanced PAC associations is assumed to be due to *de novo* mutations in male germ line cells. Whereas female germ cells undergo a total of 23 chromosomal replications, all of which take place prenatally, in males, spermatogenesis continues during most of men’s lifetime thus increasing the possibility of random mutations with age [13]. It has been estimated that by the age of 20 there has been approximately 150 replications in male germ line cells, whereas by the age of 40 the number can be as high as 610 [13]. Other sources estimate that each year PAC increases the offspring *de novo* mutations by 4% [14]. In addition to mutations, epigenetic modifications, such as DNA methylation and histone tail modifications, are plausible candidates behind advanced PAC. During spermatogenesis and spermiogenesis, the germ cell DNA undergoes heavy epigenetic reprogramming that can be easily disrupted [15]. Generally, it is thought that the methylation of DNA and histone modifications are functioning in transcriptional silencing and the effects are transmitted through altered gene expression. It has been shown that the levels of 5-methylcytosise and 5-hydroxymethylcytosine increased with advancing PAC [16].

Delayed maternal age at conception (MAC) has been associated with restricted intrauterine growth and reduced birth weight and it has also been shown to increase the risk of congenital malformations and perinatal mortality [17]. Most of the undesirable effects associated with the advanced maternal age at conception, such as Down syndrome, stem from increased incidences of chromosomal abnormalities [18].

Menopause defines a time point after which women are biologically incapable to reproduce and experience a notable decrease in oocyte production. However, men are traditionally not considered to be limited by this type of fertility window [19]. Age-wise, men are always fertile as the production of male germ cells continues throughout their lifetime. As previously noted, the effects of advanced maternal age at conception on offspring are well known, whereas the corresponding information concerning advanced paternal age at conception is lacking. To address this, aspects such as the quality of sperm, reproductive success and effects on offspring have been studied. The effects of age on semen quality has been a topic of controversy; it has been noted that there is no age-associated decline in the quality of semen [20]. However, the latest systematic review of the 90 previous studies states that while there is no notable decline in the concentration of sperm, the motility of sperm decreases and DNA fragmentation increases [21]. In addition, there is evidence that reproductive success is lowered with advanced PAC [22].

The effects of advanced PAC on the health of the offspring has been frequently studied lately; however, the data available from genome-wide association studies are still lacking. These data are necessary to fully understand the mechanisms and implications of male reproductive aging. The aim of this study was to identify genome-wide transcriptomic implications associated with advanced PAC in the blood cells of 90 year old individuals to see if increase in PAC could have impact in the aging process.

## Materials and Methods

### Study Population

The study population was comprised of 34 nonagenarian individuals (women n = 25, men n = 13) who also participated in the Vitality 90+ study, a prospective population-based study focusing on individuals aged 90 years and older living in the city of Tampere, Finland. All participants in the study were of Western European descent (for a more detailed description of recruitment and characterization of the participants see [23]. Each study participant agreed to written, informed consent and the guidelines of the Declaration of Helsinki were followed in the study protocol. The study protocol was approved by the ethics committee of the city of Tampere (Study protocol number SOTE 1592/402/96). This study involved individuals born in 1920, with biological samples collected in 2010, at which time the participants were 90 years old [24]. The final number of the select 34 nonagenarians was dictated by the availability of data concerning the ages of their fathers at the time of conception in the local parish register.

### Sample Collection

The sample collection was performed as described previously in [25]. The blood draw was performed by trained medical students during home visits and samples were drawn into EDTA tubes between 8 a.m. and 12 a.m. Leukocyte separation was applied directly on fresh blood samples using a Ficoll-Paque density gradient (Ficoll-Paque^™^ Premium, cat no. 17-5442-03, GE Healthcare Bio-Sciences AB, Uppsala, Sweden). The obtained PBMC layers were collected, and a subset of these cells was stored in RNA*later* solution (Ambion Inc., Austin TX, USA) for later use in gene expression analysis.

### RNA Extraction

RNA extraction was performed as described previously in [25]. Equal amounts of PBS and RNAlater were added to PBMC suspensions to initiate RNA recovery. Then, all fluids were removed by centrifugation, yielding only the cell pellets. For the actual RNA extraction, the miRNeasy mini kit (Qiagen, CA, USA) was used, according to the manufacturer’s protocol, using on-column DNase digestion (AppliChem GmbH, Darmstadt, Germany). Finally, the quality and concentration of the RNA were assessed using an Agilent RNA 6000 Nano Kit on an Agilent 2100 Bioanalyzer (Agilent Technologies, CA, USA).

### Expression Array

Expression arrays were prepared and analyzed as described previously in [25]. A total of 330 ng of RNA was used to prepare labeled complementary RNA (cRNA) with the Illumina TotalPrep RNA Amplification Kit (Ambion Inc., TX, USA), according to the manufacturer’s protocol with an overnight incubation. The 2100 Bioanalyzer (Agilent Technologies) was used to determine the quality and concentration of the obtained cRNA. The samples were hybridized to HumanHT-12 v4 expression BeadChip (cat. no. BD-103-0204, Illumina Inc., CA, USA) according to the Illumina protocol using 1500 ng of labeled cRNA for each sample. The experiment was performed at the Department of Biotechnology, University of Tartu. The samples were applied randomly on the BeadChips and scanning was performed with BeadScan (Illumina Inc., CA, USA).

### Preprocessing of Gene Expression Microarray Data

Gene expression microarray data was preprocessed as described previously in [25]. The gene expression microarray data were preprocessed using the R software as a *Lumibatch* object and with the *lumi* pipeline [26]. The bgAdjust.affy package was applied for the background correction and gene expression values were *log2* transformed followed by normalization with the *rsn* method. Only the probes with log2-transformed expression values between 5 and 100 were included to dispose of the non-expressed probes and bad-quality data. Visualization tools such as boxplots and principal component analysis (PCA) were used to verify the quality of the data (See S1 Fig for the PCA plots of the normalized gene expression data assorted by gender and experimental batch information).

### Determination of Paternal Age at Conception and Its Correlation with Gene Expression

The local parish register provided the birth data for the fathers of the nonagenarians. Together, with the birth data for the nonagenarians, this information was used to calculate the age of these fathers at the time when their offspring were conceived. This age, the paternal age at conception, was then correlated with the gene expression data using bivariate correlation (Spearman) to identify the transcripts that were associated with this particular variable. The applied cut-off level for significance was set to FDR < 20%. The observed correlations were assessed by performing permutation tests (S7 Table).

### Confounding Factors

Maternal age at conception (MAC) correlated significantly with PAC (p<0.005, r = 0.9), and could thus not be used as an adjusting factor. To address this, gene expression profile was correlated with MAC using bivariate correlation (Spearman) with the significance cut-off level of FDR < 20%. The effect of gender was also taken into consideration; the correlation analysis between PAC and gene expression was checked separately in men and women.

### Pathway Analyses

The identified transcripts, associated with PAC were analyzed for potential pathway enrichment in gene ontology. Two dedicated online tools were used to accomplish this: Gene Ontology enrichment analysis and visualization tool [27] and Ingenuity Pathway Analysis (IPA^®^, QIAGEN Redwood City, www.qiagen.com/ingenuity, [28]). GOrilla was utilized to identify the possible enriched GO cellular processes and components associated with PAC. The analysis was carried out in the *two unranked lists of genes*–mode that compares the given set of transcripts to a background set. In this case, the target set consisted of the identified PAC associated 648 transcripts and the background set consisted of all the possible transcripts in the used expression array. The p-value threshold was set to 10E-7 to exclude a portion of the low-significance GO terms that were numerous, and this corresponded to a Benjamini-Hochberg multiple test correction p-value of 0.05. Duplicated or unresolved transcripts were not included in the run.

IPA software (IPA^®^, QIAGEN Redwood City [28]) was used to look for the enrichment of transcripts in canonical pathways. All data sources provided by the Ingenuity Knowledge Base were included and also used a reference set for analysis. Only data from humans were considered and results for the molecular associations were required to be experimentally observed. The canonical pathway p-values were Benjamini-Hochberg multiple testing corrected and to be considered significant, a pathway was required to contain at least three genes.

### Array Data

The gene expression microarray data are available in the GEO database (http://www.ncbi.nlm.nih.gov/geo/) GSE40366.

## Results

The study population consisted of 38 nonagenarian individuals (women n = 25, men n = 13) whose peripheral blood mononuclear cell (PBMC) gene expression data were available. The mean PAC of the nonagenarians was 35.05 years (minimum = 21, maximum = 58, std. deviation = 7.64). Statistical analysis revealed a total of 648 transcripts in which the level of gene expression correlated with PAC (FDR < 20%) (See S1 Table for the full list of transcripts). Approximately half of the transcripts (n = 348, 54% of total) were down-regulated with increasing paternal age at conception and 300 transcripts (46% of total) were up-regulated. The gene with the most significant positive association with PAC was *AHSA1* (Benjamini-Hochberg corrected p = 0.047, R = 0.637), which codes for chaperone protein that regulates heat shock protein 90. The most significant negative association was found with *CHCHD5* (Benjamini-Hochberg corrected p = 0.047, R = -0.658), coding a mitochondrial intermembrane space protein, which operates to promote keeping the mitochondrial substrates in their respective compartments [29]. The top 20 associated transcripts are listed in Table 1.

**Table 1:** The top 20 transcripts that were associated with paternal age at conception. The table shows gene, gene description, correlation coefficient R (Spearman’s Rho), non-adjusted p-value and Benjamini-Hochberg-adjusted p-value (FDR).

Gene	Description	R	p-value	FDR
*CHCHD5*	coiled-coil-helix-coiled-coil-helix domain containing 5	-0,658	7,09E-06	0,047
*PILRA*	paired immunoglobin-like type 2 receptor alpha	-0,638	1,61E-05	0,047
*AHSA2*	AHA1, activator of heat shock 90kDa protein ATPase homolog 2 (yeast)	0,637	1,69E-05	0,047
*RNF5P1*	ring finger protein 5, E3 ubiquitin protein ligase pseudogene 1	-0,636	1,79E-05	0,047
*VMA21*	VMA21 vacuolar H+-ATPase homolog (S, cerevisiae)	0,627	2,53E-05	0,054
*NA*	NA	-0,613	4,26E-05	0,075
*HSD17B10*	hydroxysteroid (17-beta) dehydrogenase 10	-0,598	7,36E-05	0,106
*NOL8*	nucleolar protein 8	0,592	9,00E-05	0,106
*NA*	NA	-0,587	1,07E-04	0,106
*PSMB5*	proteasome (prosome, macropain) subunit, beta type, 5	-0,585	1,16E-04	0,106
*MRPL14*	mitochondrial ribosomal protein L14	-0,583	1,22E-04	0,106
*C1orf63*	chromosome 1 open reading frame 63	0,581	1,29E-04	0,106
*FOXJ3*	forkhead box J3	0,581	1,30E-04	0,106
*SLU7*	SLU7 splicing factor homolog (S, cerevisiae)	0,579	1,41E-04	0,107
*SEC13*	SEC13 homolog (S, cerevisiae)	-0,577	1,51E-04	0,107
*NHP2*	NHP2 ribonucleoprotein	-0,573	1,70E-04	0,112
*CLTA*	clathrin, light chain A	-0,568	2,02E-04	0,112
*MRPL53*	mitochondrial ribosomal protein L53	-0,567	2,08E-04	0,112
*UBE3A*	ubiquitin protein ligase E3A	0,566	2,13E-04	0,112

The 648 PAC-associated transcripts were analyzed with the GOrilla web tool in a two unranked list fashion (target versus background lists) to look for the enrichment of genes in cellular processes. GOrilla analysis revealed a total of 29 enriched processes (See Table 2) at the threshold of Benjamini-Hochberg corrected p = 5E10-5. The most significant processes were general unspecific cellular processes such as *Cellular metabolic process* and *Cellular nitrogen compound metabolic process*. This, however, is the intended tendency of this analysis tool and meaningful processes have to be looked for among the lower significances. These identified processes included *Mitochondrial translational termination* (Benjamini-Hochberg corrected p = 1.39E-5), *Antigen processing and presentation of peptide antigen via MHC class I* (Benjamini-Hochberg corrected p = 3.77E-5) and *Proteasomal ubiquitin-independent protein catabolic process* (Benjamini-Hochberg corrected p = 4.64E-5). The *Mitochondrial translational termination* term consisted of 15 mitochondrial ribosomal proteins that were all down-regulated except for one (Table 3). Furthermore, the associated 648 transcripts were analyzed by cellular component, to specify which cellular location contained the identified transcripts. This approach revealed that genes targeted for mitochondria were the most represented, namely 78 out of 648 transcripts (12% of the total).

**Table 2:** Analysis of Gene Ontology in transcripts that were associated with paternal age at conception. The table shows GO term, GO term description, p-value and Benjamini-Hochberg corrected p-value.

GO term	Description	P-value	FDR q-value
GO:0044237	cellular metabolic process	4.49E-14	6.09E-10
GO:0008152	metabolic process	9.78E-13	6.64E-9
GO:0034641	cellular nitrogen compound metabolic process	3.22E-12	1.46E-8
GO:0044238	primary metabolic process	5.55E-12	1.88E-8
GO:0044260	cellular macromolecule metabolic process	2.5E-11	6.78E-8
GO:0016071	mRNA metabolic process	4.82E-11	1.09E-7
GO:0071704	organic substance metabolic process	6.58E-11	1.28E-7
GO:0006807	nitrogen compound metabolic process	2.58E-10	4.37E-7
GO:0006415	translational termination	2.59E-10	3.91E-7
GO:0006397	mRNA processing	4.33E-10	5.88E-7
GO:0006396	RNA processing	1.91E-9	2.36E-6
GO:0008380	RNA splicing	1.95E-9	2.2E-6
GO:0043170	macromolecule metabolic process	2.77E-9	2.9E-6
GO:0006412	translation	4.75E-9	4.6E-6
GO:0043043	peptide biosynthetic process	5.34E-9	4.83E-6
GO:0043624	cellular protein complex disassembly	6.19E-9	5.25E-6
GO:0043241	protein complex disassembly	9.14E-9	7.3E-6
GO:0010467	gene expression	1.32E-8	9.93E-6
GO:0032984	macromolecular complex disassembly	1.74E-8	1.25E-5
GO:0070126	mitochondrial translational termination	2.05E-8	1.39E-5
GO:0006518	peptide metabolic process	4.93E-8	3.19E-5
GO:0002474	antigen processing and presentation of peptide antigen via MHC class I	6.12E-8	3.77E-5
GO:0006139	nucleobase-containing compound metabolic process	6.21E-8	3.66E-5
GO:0000377	RNA splicing, via transesterification reactions with bulged adenosine as nucleophile	7.25E-8	4.1E-5
GO:0000398	mRNA splicing, via spliceosome	7.25E-8	3.94E-5
GO:0043604	amide biosynthetic process	8.71E-8	4.54E-5
GO:0090304	nucleic acid metabolic process	9.55E-8	4.8E-5
GO:0016070	RNA metabolic process	9.67E-8	4.69E-5
GO:0010499	proteasomal ubiquitin-independent protein catabolic process	9.91E-8	4.64E-5

**Table 3:** The associated transcripts inside the GO term *Mitochondrial translational termination*. All but one are exclusively down-regulated. Table shows gene name, gene description, correlation coefficient (Spearman’s Rho) and Benjamini-Hochberg-adjusted p-value.

Gene	Description	R	p-value
*MRPL14*	mitochondrial ribosomal protein L14	-0.583	0.106
*MRPL53*	mitochondrial ribosomal protein L53	-0.567	0.112
*MRPS11*	mitochondrial ribosomal protein S11	-0.520	0.144
*MTRF1*	mitochondrial translational release factor 1	0.515	0.144
*MTRF1L*	mitochondrial translational release factor 1-like	-0.490	0.148
*MRPS12*	mitochondrial ribosomal protein S12	-0.465	0.163
*MRPL21*	mitochondrial ribosomal protein L21	-0.457	0.165
*MRPS18A*	mitochondrial ribosomal protein S18A	-0.454	0.167
*MRPS34*	mitochondrial ribosomal protein S34	-0.448	0.169
*MRPL11*	mitochondrial ribosomal protein L11	-0.438	0.172
*MRPS23*	mitochondrial ribosomal protein S23	-0.439	0.172
*MRPS7*	mitochondrial ribosomal protein S7	-0.423	0.178
*MRPL23*	mitochondrial ribosomal protein L23	-0.418	0.181
*MRPL33*	mitochondrial ribosomal protein L33	-0.405	0.196
*MRRF*	mitochondrial ribosome recycling factor	-0.404	0.197

Further inspection of the relationships between the identified transcripts in the IPA web tool revealed six significant (Benjamini-Hochberg-corrected p-value <0.05) canonical pathways. These pathways were *Mitochondrial Dysfunction*, *Systemic Lupus Erythematosus Signaling*, *Oxidative Phosphorylation*, *Protein Ubiquitination Pathway*, *Antigen Presentation Pathway* and the *Superpathway of Cholesterol Biosynthesis*. Specifically, the *Mitochondrial Dysfunction* and *Oxidative Phosphorylation* pathways were of interest because mitochondria were also identified in the GOrilla analysis.

**Fig 1:**
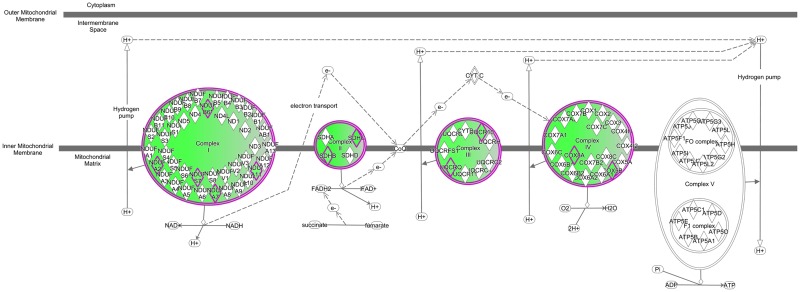
Affected *Oxidative Phosphorylation* pathway. The down-regulation of the genes in *Oxidative Phosphorylation* pathway is associated with increased PAC. The identified transcripts were components of complexes I, II, III and IV of the respiratory chain in mitochondria. Transcript ATP5LΔ in complex V is not shown. The figure was produced using Ingenuity Pathway Analysis tool.

The *Oxidative Phosphorylation* pathway included 12 associated transcripts. These were: *ATP5LΔ*, *COX5B*, *COX8A*, *NDUFA2*, *NDUFA7*, *NDUFA11*, *NDUFB6*, *NDUFS7*, *SDHB*, *SDHC*, *UQCR10* and *UQCRQ* (See Table 4 for descriptions of the *Oxidative Phosphorylation* pathway genes) and they were exclusively down-regulated with increasing paternal age at conception (Fig 1). All these transcripts function as components of complexes I-V in the mitochondrial respiratory chain. The *Mitochondrial Dysfunction* pathway included all the transcripts in the *Oxidative Phosphorylation* pathway and additional genes that function in the context of oxidative stress and the citric acid cycle. These included *PARK7*, *CAT*, *ABAD*, *PRX5* and *ACO2*, and were also consistently down-regulated with increasing PAC (See Table 5 for descriptions of the *Mitochondrial Dysfunction* pathway genes). Correlation analysis of MAC with gene expression profile identified 213 associated transcripts (S2 Table). No enriched pathways could be identified with GOrilla or IPA in the case of MAC associated transcripts. The 648 PAC associated and 213 MAC associated transcripts included 46 common genes (S3 Table). These transcripts did not include the mitochondrial dysfunction associated genes observed in the case of PAC, suggesting that this effect is associated mainly to paternal age at conception.

**Table 4:** The PAC-associated transcripts identified with IPA in the Oxidative phosphorylation canonical pathway. Table shows symbol, Entrez gene name and correlation coefficient, R.

Symbol	Entrez Gene Name	R
*ATP5L*	ATP Synthase, H+ Transporting, Mitochondrial Fo Complex Subunit G	-0.505
*COX5B*	cytochrome c oxidase subunit Vb	-0.499
*COX8A*	cytochrome c oxidase subunit VIIIA (ubiquitous)	-0.511
*NDUFA2*	NADH dehydrogenase (ubiquinone) 1 alpha subcomplex, 2, 8kDa	-0.511
*NDUFA7*	NADH dehydrogenase (ubiquinone) 1 alpha subcomplex, 7, 14.5kDa	-0.495
*NDUFA11*	NADH dehydrogenase (ubiquinone) 1 alpha subcomplex, 11, 14.7kDa	-0.410
*NDUFB6*	NADH dehydrogenase (ubiquinone) 1 beta subcomplex, 6, 17kDa	-0.494
*NDUFS7*	NADH dehydrogenase (ubiquinone) Fe-S protein 7, 20kDa (NADH-coenzyme Q reductase)	-0.431
*SDHB*	succinate dehydrogenase complex, subunit B, iron sulfur (Ip)	-0.448
*SDHC*	succinate dehydrogenase complex, subunit C, integral membrane protein, 15kDa	-0.548
*UQCR10*	ubiquinol-cytochrome c reductase, complex III subunit X	-0.461
*UQCRQ*	ubiquinol-cytochrome c reductase, complex III subunit VII, 9.5kDa	-0.444

**Table 5:** The PAC-associated transcripts identified with IPA in the Mitochondrial dysfunction canonical pathway. Table shows symbol, Entrez gene name and correlation coefficient, R.

Symbol	Entrez Gene Name	R
*ACO2*	aconitase 2, mitochondrial	-0.404
*ATP5L*	--	-0.505
*CAT*	catalase	-0.403
*COX5B*	cytochrome c oxidase subunit Vb	-0.499
*COX8A*	cytochrome c oxidase subunit VIIIA (ubiquitous)	-0.511
*HSD17B10*	hydroxysteroid (17-beta) dehydrogenase 10	-0.598
*NDUFA2*	NADH dehydrogenase (ubiquinone) 1 alpha subcomplex, 2, 8kDa	-0.511
*NDUFA7*	NADH dehydrogenase (ubiquinone) 1 alpha subcomplex, 7, 14.5kDa	-0.495
*NDUFA11*	NADH dehydrogenase (ubiquinone) 1 alpha subcomplex, 11, 14.7kDa	-0.410
*NDUFB6*	NADH dehydrogenase (ubiquinone) 1 beta subcomplex, 6, 17kDa	-0.494
*NDUFS7*	NADH dehydrogenase (ubiquinone) Fe-S protein 7, 20kDa (NADH-coenzyme Q reductase)	-0.431
*PARK7*	parkinson protein 7	-0.459
*PRDX5*	peroxiredoxin 5	-0.524
*SDHB*	succinate dehydrogenase complex, subunit B, iron sulfur (Ip)	-0.448
*SDHC*	succinate dehydrogenase complex, subunit C, integral membrane protein, 15kDa	-0.548
*UQCR10*	ubiquinol-cytochrome c reductase, complex III subunit X	-0.461
*UQCRQ*	ubiquinol-cytochrome c reductase, complex III subunit VII, 9.5kDa	-0.444

When the correlation analysis between PAC and gene expression was performed separately in men and women, the earlier observed effect was not seen as clearly. In the case of women (n = 25) the correlation coefficients were generally low and significances high (S4 Table). The highest observed FDR observed in women was as high as 0.796. On the other hand, correlation coefficients seen in the case of men (n = 13) were relatively much higher and significances lower, although only 17 transcripts were below FDR 0.2 (S5 Table). To get the better idea of the possible effect of gender we picked the previously identified transcripts within the whole study population, in the *Mitochondrial dysfunction pathway* and compared their correlation coefficients and significances separately in men and women (S6 Table). This comparison suggests that male individuals could be more prone to the effects of increased paternal age at conception, as the correlation coefficients are relatively higher in the male individuals.

## Discussion

The human race is facing an interesting transition, where more and more individuals deliberately decide to delay their onset of reproduction [1]. One might argue that this trend in advancing parental ages puts us in unconventional circumstances because our bodies have most likely been selected for reproduction at an earlier age, right after sexual maturity. Therefore, it is not surprising that delaying the period of reproduction could give rise to undesired effects in offspring. The possible effects of advanced PAC in offspring has stirred up public discussion; however, this discussion is less than that regarding advanced maternal age, and rightfully so. Although the observed effects of advanced PAC are not overly pronounced, they are definitely worth identifying. Studies that have contributed to investigating the adverse effects of advanced PAC have mainly focused on phenotypic manifestations of associated diseases. The goal of this study was to investigate the effects of advanced PAC on offspring at the level of gene expression. By accomplishing this in very old individuals we were able to evaluate the effects of increased PAC in the aging process.

Our main finding was that advanced PAC was associated with the small-scale down-regulation of a variety of nuclear-coded mitochondrial genes in their 90-year old offspring. More specifically, genes functioning in the mitochondrial respiratory chain and mitochondrial translation machinery were affected. Down-regulation of components in the respiratory chain is usually a manifestation of compromised function in oxidative phosphorylation and therefore, energy metabolism. It has been shown that aging is associated with down-regulation of mitochondrial respiratory chain genes in mice and this causality is mainly attributed to mtDNA mutations [30]. Therefore, one might argue, if the observed effect is not associated with calendar age but with the biological aging instead. Mitochondrial DNA mutations could be the underlying cause that leads to the changes in the transcription of nuclear coded mitochondrial genes.

In addition, impairments in mitochondrial translational machinery, which is needed in the assembly of the proteins coded by mitochondrial genome, can predispose to mitochondrial diseases [31]. Down-regulation of two separate mitochondrial processes were observed, the respiratory chain and translational functions, which might be related to each other in coordination of both nuclear and mitochondrial genetic systems that are needed to establish proper mitochondrial function [32]. However, down-regulation of both the mitochondrial respiratory chain and translational machinery components suggest that advanced PAC is associated with dysfunctional mitochondria in the offspring. Mitochondrial dysfunction is often implicated in the aging process and one of the main aging theories underlines the importance of free radicals in aging [33]. Mitochondrial dysfunction can give rise to reactive oxygen species or the mitochondria can become dysfunctional resulting from increased oxidative stress. The observed down-regulation observed in our results could be from cellular adjustment to increased oxidative stress. How, then, could increased PAC contribute to oxidative stress or mitochondrial dysfunction? *De novo* mutations in paternal germline cells are proposed to be the main mode of transmission for the advanced PAC effect. It is unlikely that random mutations in sperm DNA would take place in nuclear-coded mitochondrial respiratory chain genes because these do not seem to be mutational hotspots. In contrast, mitochondria-coded genes are more prone and more likely to accumulate mutations due to oxidative stress generated in mitochondria [34]. It has also been shown that oxidative stress induces mitochondrial dysfunction in cell lines [35], which supports the idea that oxidative stress could be the ultimate driving force behind advanced PAC. Unfortunately, we had no opportunity to measure cellular markers of oxidative stress in these individuals to confirm this idea.

Maternal age at conception correlated heavily with PAC and thus, could not be used as adjusting factor. We addressed this by checking gene expression correlation with MAC and did not observe the same results. MAC was associated with differential expression of 213 transcripts, but these could not be tracked to any specific cellular pathways of processes. Additionally, 46 transcripts were common for the 648 PAC associated and 213 MAC associated transcripts, but they were not those identified in mitochondrial dysfunction processes and pathways, our main finding. Thus, we conclude that observed PAC-associated pathways are indeed affected by increased PAC and not increased MAC.

Based on the gender-specific analysis of association between PAC and the PBMC gene expression, it appears that PAC could have larger effect specifically on the male individuals. The gender-specific comparison of the genes identified in the *Mitochondrial dysfunction pathway* (Table 5) in the whole study population suggests that the effect might be more prominent in men (S7 Table). However, as the number on samples becomes really small (n = 13 for men and n = 25 for women), the correlation coefficients and significances of correlation analysis become more difficult to interpret. Therefore, we cannot claim that the gender effect is significant and this aspect has to be investigated with larger study populations.

An important question to consider is whether the genetic mutations can be solely responsible for the observed PAC associated effects. A previous study found that the spontaneous mutation rate in male germ cells was not necessarily high enough to account for these effects [36], therefore suggesting that other mechanisms could be involved. In light of this idea of transgenerational epigenetic effects, it is tempting to speculate the involvement of epigenetic mechanisms as contributors to advanced PAC effects.

In conclusion, we found that advanced paternal age at conception is associated with mitochondrial dysfunction manifested by small-scale down-regulation of the genes involved in mitochondrial translation and the respiratory chain, which was analyzed in PBMCs of nonagenarian individuals. Mitochondrial dysfunction is assumed to give rise to oxidative stress and the observed uniform down-regulation of respiratory chain complex genes could indeed result from excess oxidative stress produced in the oxidative phosphorylation. Advanced PAC has been linked to various adverse effects in offspring, such as detrimental birth outcomes and pregnancy effects, and mental illnesses. However, the study population was considered healthy in this respect. This suggests that the mechanism exerted by advanced PAC might act in threshold-like manner. Therefore, we can see some changes at the gene expression level of these nonagenarian individuals, even though they are phenotypically healthy, i.e., not suffering from the clinically described effects from advanced PAC. Interestingly, the pathophysiology of mental illnesses, such as schizophrenia and autism, are often linked to mitochondrial dysfunction, which could partially explain the association between mental illnesses and advanced PAC. Based on our results, advanced PAC could have a partial contribution to the pathophysiology of these neurological disorders, through mitochondrial defects.

## Supporting Information

S1 Fig **S1A Fig**. Principal component analysis plot of the normalized gene expression data from PBMC:s of the nonagenarian individuals. Here are shown principal components 1–3. PC1 (x-axis) explains 17%, PC2 (y-axis) 12.4% and PC3 (z-axis) 9.1% of the total variance. Colors indicate experimental batches. **S1B Fig**. Principal component analysis plot of the normalized gene expression data from PBMC:s of the nonagenarian individuals. Here are shown principal components 4–6. PC4 (x-axis) explains 6.1%, PC5 5.5% and PC6 4.5% of the total variance. Colors indicate experimental batches. **S1C Fig**. Principal component analysis plot of the normalized gene expression data from PBMC:s of the nonagenarian individuals. Here are shown principal components 1–3. PC1 (x-axis) explains 17%, PC2 (y-axis) 12.4% and PC3 (z-axis) 9.1% of the total variance. Colors indicate gender. **S1D Fig**. Principal component analysis plot of the normalized gene expression data from PBMC:s of the nonagenarian individuals. Here are shown principal components 4–6. PC4 (x-axis) explains 6.1%, PC5 5.5% and PC6 4.5% of the total variance. Colors indicate gender.
(DOCX)

S1 Table Correlation analysis of paternal age at conception (PAC) and PBMC gene expression. **PAC was associated with expression of 648 transcripts (FDR < 20%)**. nuIlID is a unique identifier for the probes used in the HumanHT-12 beadchip provided by Illumina. FDR is false discovery rate.
(XLSX)

S2 Table Correlation analysis of maternal age at conception (MAC) and PBMC gene expression. **MAC was associated with expression of 213 transcripts (FDR < 20%)**. nuIlID is a unique identifier for the probes used in the HumanHT-12 beadchip provided by Illumina. FDR is false discovery rate.
(XLSX)

S3 Table A total of 46 common transcripts were identified to be associated with both PAC and MAC. nuIlID is a unique identifier for the probes used in the HumanHT-12 beadchip provided by Illumina.
(XLSX)

S4 Table Correlation analysis of paternal age at conception (PAC) and PBMC gene expression of nonagenarian female individuals. nuIlID is a unique identifier for the probes used in the HumanHT-12 beadchip provided by Illumina. FDR is false discovery rate.
(XLSX)

S5 Table Correlation analysis of paternal age at conception (PAC) and PBMC gene expression in nonagenarian male individuals. nuIlID is a unique identifier for the probes used in the HumanHT-12 beadchip provided by Illumina. FDR is false discovery rate.
(XLSX)

S6 Table Gender-specific comparison of the correlations between PAC and gene expression in the Mitochondrial dysfunction pathway transcripts identified in analysis carried out with total population. nuIlID is a unique identifier for the probes used in the HumanHT-12 beadchip provided by Illumina. FDR is false discovery rate.
(XLSX)

S7 Table Assessment of the observed correlations using permutation tests. Spearman's Rho, p-value and FDR columns refer to the results obtained by correlating PAC and PBMC gene expression. For the permutation tests, sample population was divided to the individuals having older fathers and those having younger fathers. Mean young and Mean old columns present the mean expression values for these two groups for each transcript. Fold change and Log2 fold change indicate the respective fold change of the expression between the two groups. Empirical p-value indicates the p-value that was obtained by performing 10000 resampling permutations with randomization of the two groups.
(XLSX)
