# Supplementary material for: Increased Paternal Age at Conception Is Associated with Transcriptomic Changes Involved in Mitochondrial Function in Elderly Individuals
Source: PLoS One. 2016 Nov 23;11(11):e0167028. doi: 10.1371/journal.pone.0167028 (PMC5120832; doi:10.1371/journal.pone.0167028)
Supplement: S1 Fig — S1A Fig. Principal component analysis plot of the normalized gene expression data from PBMC:s of the nonagenarian individuals. Here are shown principal components 1–3. PC1 (x-axis) explains 17%, PC2 (y-axis) 12.4% and PC3 (z-axis) 9.1% of the total variance. Colors indicate experimental batches. S1B Fig. Principal component analysis plot of the normalized gene expression data from PBMC:s of the nonagenarian individuals. Here are shown principal components 4–6. PC4 (x-axis) explains 6.1%, PC5 5.5% and PC6 4.5% of the total variance. Colors indicate experimental batches. S1C Fig. Principal component analysis plot of the normalized gene expression data from PBMC:s of the nonagenarian individuals. Here are shown principal components 1–3. PC1 (x-axis) explains 17%, PC2 (y-axis) 12.4% and PC3 (z-axis) 9.1% of the total variance. Colors indicate gender. S1D Fig. Principal component analysis plot of the normalized gene expression data from PBMC:s of the nonagenarian individuals. Here are shown principal components 4–6. PC4 (x-axis) explains 6.1%, PC5 5.5% and PC6 4.5% of the total variance. Colors indicate gender. (DOCX) [file pone.0167028.s001.docx]

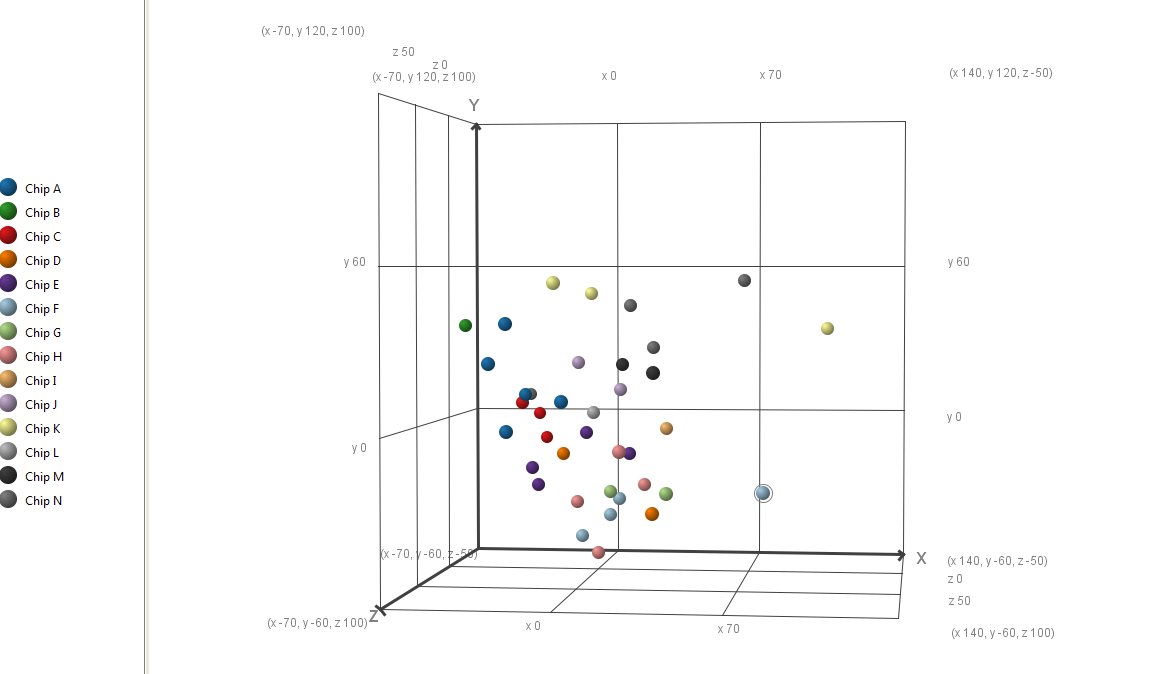


S1A Figure. Principal component analysis plot of the normalized gene expression data from PBMC:s of the nonagenarian individuals. Here are shown principal components 1-3. PC1 (x-axis) explains 17 %, PC2 (y-axis) 12.4 % and PC3 (z-axis) 9.1 % of the total variance. Colors indicate experimental batches.


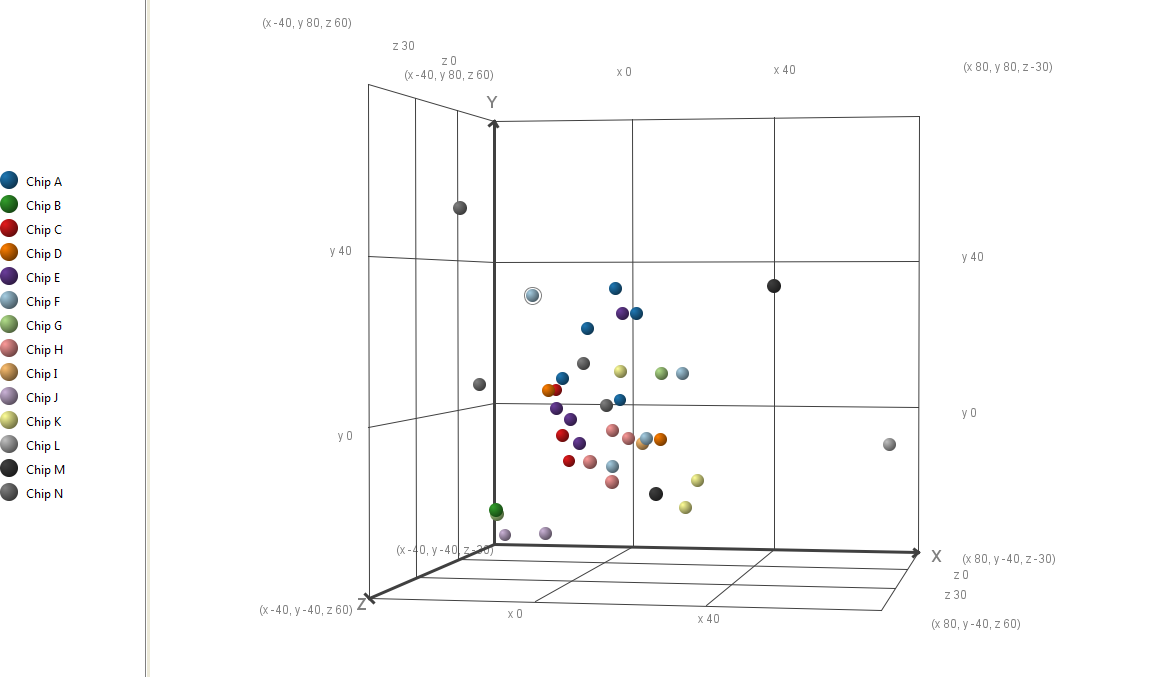


S1B Figure. Principal component analysis plot of the normalized gene expression data from PBMC:s of the nonagenarian individuals. Here are shown principal components 4-6. PC4 (x-axis) explains 6.1 %, PC5 5.5% and PC6 4.5 % of the total variance. Colors indicate experimental batches.


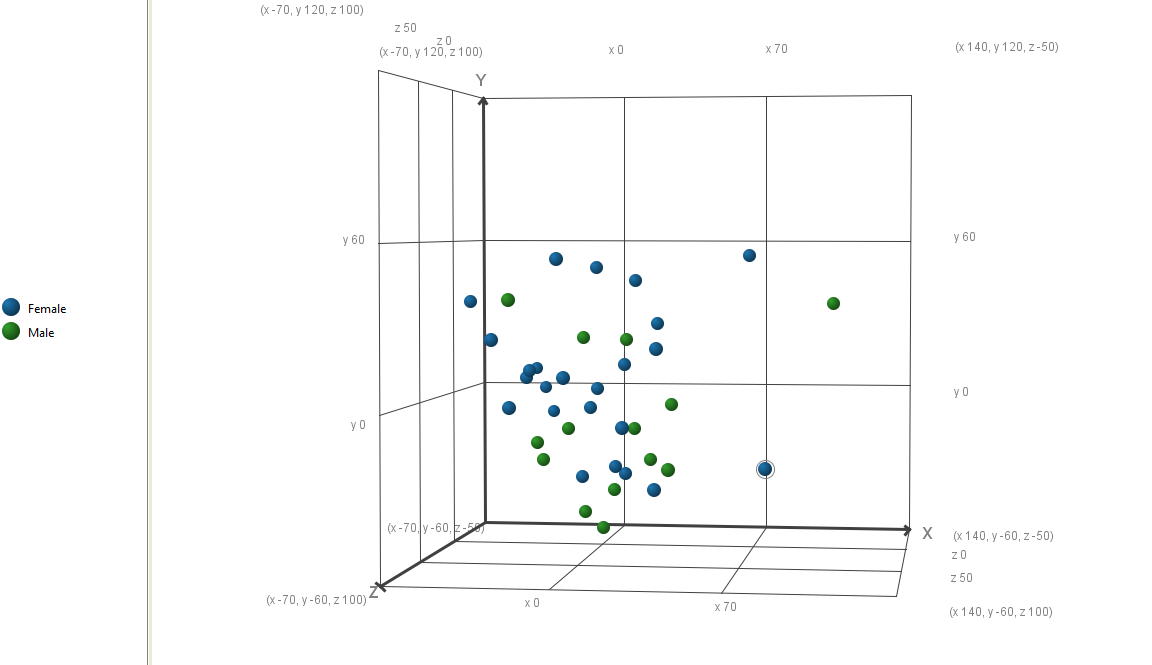


S1C Figure. Principal component analysis plot of the normalized gene expression data from PBMC:s of the nonagenarian individuals. Here are shown principal components 1-3. PC1 (x-axis) explains 17 %, PC2 (y-axis) 12.4 % and PC3 (z-axis) 9.1 % of the total variance. Colors indicate gender.


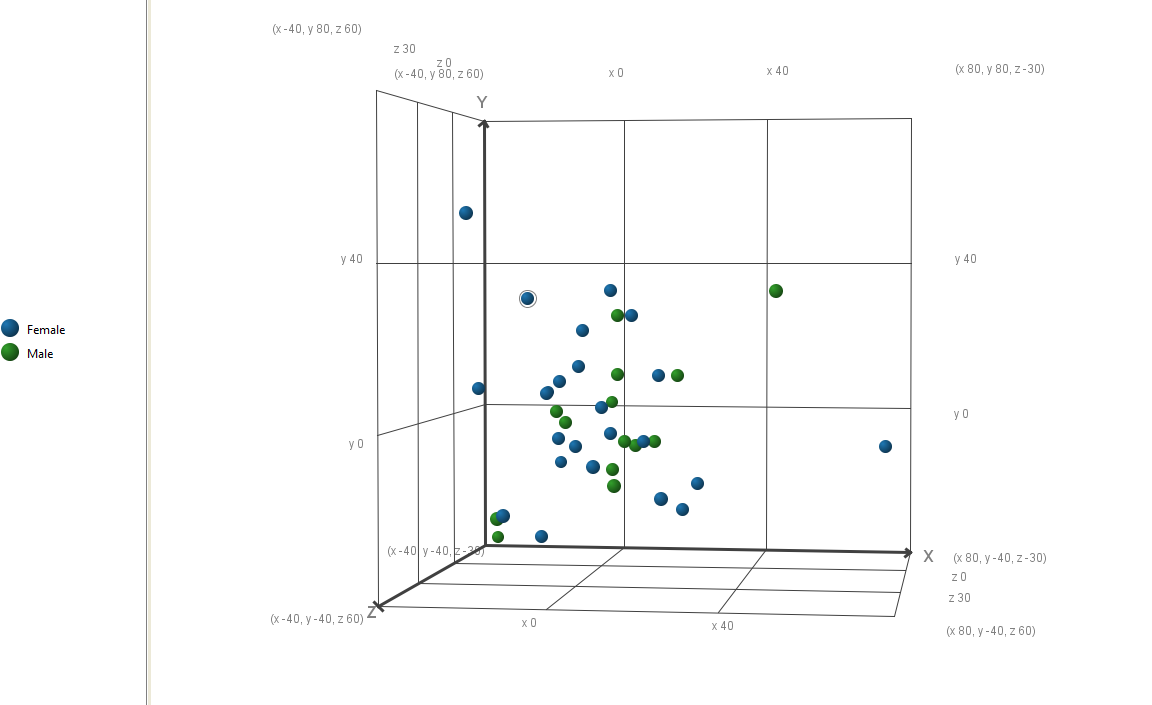


S1D Figure. Principal component analysis plot of the normalized gene expression data from PBMC:s of the nonagenarian individuals. Here are shown principal components 4-6. PC4 (x-axis) explains 6.1 %, PC5 5.5% and PC6 4.5 % of the total variance. Colors indicate gender.
